# Vitamin D is inversely associated with Monocyte to HDL-C ratio among medical staff in Chengdu, China

**DOI:** 10.1186/s12902-023-01406-2

**Published:** 2023-07-12

**Authors:** Lei Tang, Honglian Zeng, Bo Yang, Chaohui Dong, Mao Li, Xiaoli Zhang, Jia Pan

**Affiliations:** Department of Health Management Center, Affiliated Hospital of Chengdu University, Chengdu, Sichuan China

**Keywords:** Vitamin D, HDL, Monocyte percentage, MHR

## Abstract

**Purpose:**

The primary objective of this study was to explore the association of vitamin D with the monocyte to HDL-C ratio (MHR) among medical staff in Chengdu, China.

**Materials and methods:**

This cross-sectional study involved 538 medical staff, including 393 females and 145 males, and included data on gender, age, body mass index, and laboratory parameters (including complete blood count, vitamin D levels, lipid, etc.). According to serum 25 (OH)D < 20 ng/ml as vitamin D deficiency, subjects were divided into two groups based on serum 25 (OH)D levels: a vitamin D deficiency group with serum 25 (OH)D < 20 ng/ml and a vitamin D sufficiency group with serum 25 (OH)D ≥ 20 ng/ml. When considering vitamin D as a categorical variable, a multivariable logistic regression analysis was conducted to assess the risk factors associated with vitamin D deficiency. On the other hand, when examining the factors influencing vitamin D levels as a continuous variable, a multiple linear regression model was utilized.

**Results:**

The prevalence of vitamin D deficiency was 86.25% among all the participants. Males exhibited a higher risk of vitamin D deficiency compared to females (*β*=0.383, *P* = 0.026). Vitamin D deficiency risk decreased with age (*β* = 0.910, *P* < 0.001). Additionally, elevated values of MHR were associated with an increased risk of vitamin D deficiency (β = 1.530, *P* = 0.019). When treating vitamin D as a continuous variable, the results of multiple linear regression revealed that age (*β* = 0.342, *P* < 0.001), and TG (*β*=-1.327, *P* = 0.010) were independent influencing factors for vitamin D levels, indicating that vitamin D levels increase with age. A reverse association between MHR and vitamin D levels demonstrated a marginal trend toward significance (β=-0.581, *P* = 0.052).

**Conclusions:**

Vitamin D is inversely associated with MHR among young medical staff in Chengdu, China.

## Introduction

25-Hydroxyvitamin D (25[OH]D), a precursor of activated vitamin D, increases in response to sunlight exposure [1]. With the development of society and changes in lifestyle, particularly reduced outdoor activity time, vitamin D deficiency has become a global public health problem [2]. Epidemiological data indicated that vitamin D deficiency was prevalent in the Chinese population [3, 4]. Medical staff, as one of the typical occupational populations engaged in indoor work, face a challenge in acquiring sufficient vitamin D through sunlight exposure.

Vitamin D is a prohormone that is considered a marker of good health when found in adequate levels in the plasma. The classical role of vitamin D is to maintain the homeostasis of calcium and phosphorous. However, vitamin D deficiency is related to other diseases, including cancer, systemic inflammation, autoimmune diseases, and diabetic complications [5–7]. The escalating cost of the vitamin D test has led to a low screening rate [8].

Since monocytes are the major secretors of pro-inflammatory mediators such as cytokines, a high percentage of monocytes may indicate subclinical inflammation [9]. High-density lipoprotein cholesterol (HDL-C), a key component of total cholesterol, has been found to possess anti-inflammatory and protective properties [10]. Studies have indicated that the monocyte to HDL-C ratio (MHR) is a simple indicator of oxidative stress and systemic inflammation [11–13]. The regulation of systemic lipid homeostasis by the gut-adipose axis, mediated by the vitamin D receptor [14], has been demonstrated. Vitamin D has been shown to play a role in inducing autophagy, and its deficiency may contribute to the impaired antimicrobial function of monocytes [15]. However, there is no established link between serum 25 (OH)D levels and MHR. Therefore, this study aims to investigate the association between serum 25 (OH)D levels and MHR among Chengdu’s medical staff.

## Materials and methods

### Study population

This cross-sectional study included 538 medical staff, including 393 females and 145 males, from the Affiliated Hospital of Chengdu University between January and December 2020. All included participants voluntarily participated in this study and signed the informed consent form. This study was approved by the Affiliated Hospital of Chengdu University.

### Inclusion and exclusion criteria

This study recruited medical staff from the Affiliated Hospital of Chengdu University who underwent comprehensive physical examinations. The inclusion criteria were as follows: patients with complete clinical data, including blood pressure, complete blood count, vitamin D levels, lipids, fasting blood glucose (FBG), and body mass index (BMI). Participants with insufficient clinical information and a history of severe diseases (e.g., renal or liver failure or malignancy) were excluded.

### Data collection

Electronic sphygmomanometers (OMRON HEM-7136; Omron Healthcare Ltd., Kyoto, Japan) were used to measure systolic blood pressure (SBP) and diastolic blood pressure (DBP). After 5 min of rest, two 1-minute interval measures were conducted with the individuals sitting. If the difference in BP values was greater than 10 mmHg, the measurement was repeated three times, with the final reading being the mean of the two closest measurements. Trained investigators measured the body’s height and weight using standard methods. BMI was calculated as weight in kilograms divided by the square of height in meters. Laboratory testing was performed by laboratory physicians from the Affiliated Hospital of Chengdu University. Following an overnight fast of at least 8 h, venous blood was collected to test blood count (including monocytes, white blood cells (WBC), lymphocytes, and neutrophils, etc.), vitamin D level, triglyceride (TG), HDL-C, low-density lipoprotein cholesterol (LDL-C), and FBG.

### Definitions of vitamin D deficiency

According to an Endocrine Society clinical practice guideline, vitamin D deficiency is defined as a 25 (OH)D level below 20 ng/ml [16].

### Statistical analysis

Continuous variables were described by mean and standard deviation (SD). Frequencies and percentages are used to represent categorical variables. Statistical analyses were performed by *t*-test and chi-square test. Univariate linear/logical regression analysis was employed to filter for potential significant variables of continuous outcomes and categorical outcomes, respectively. Variables with a *P*-value < 0.1 in the univariate analysis were subsequently included in the multivariate linear/logistic regression analysis. Multiple logistic regression modeling will be used for categorical outcomes and multiple linear regression modeling will be used for continuous variables.

Statistical analysis was performed with SPSS software (version 25.0), with the assumed statistical significance threshold of *P* < 0.05.

## Results

### Baseline characteristics

The prevalence of vitamin D deficiency was 86.25% among all the participants. There are notable disparities in the distribution of vitamin D deficiency and sufficiency among different genders. Compared with the vitamin D sufficiency group, the vitamin D deficient group had a younger average age and higher levels of lymphocyte percentage to HDL-C ratio (LHR), MHR, and neutrophil percentage to HDL-C ratio (NHR). (Table 1)

**Table 1:** Baseline characteristics of participants based on serum 25 (OH)D concentrations (n = 538)

	Vitamin D Deficiency (N = 464)	Vitamin D Sufficiency (N = 74)	*P* value
Gender			0.002*
Male (%)	136 (29.31)	9 (12.16)	
Female (%)	328 (70.69)	65 (87.84)	
Age, year	31.7 ± 0.42	42.64 ± 1.51	< 0.001*
Weight(kg)	54.94 ± 18.40	52.57 ± 16.29	0.302
BMI, kg/m^2^	22.32 ± 0.15	22.03 ± 0.30	0.625
SBP	116.95 ± 12.21	116.51 ± 11.79	0.781
DBP	71.06 ± 9.37	70.80 ± 9.28	0.830
Smoking	28 (6.03)	5 (6.76)	0.810
WBC, cells/10^9^ L	6.14 ± 0.07	5.87 ± 0.17	0.076
LHR(%)	23.40 ± 0.33	21.01 ± 0.93	0.006*
MHR (%)	3.84 ± 0.06	3.23 ± 0.12	< 0.001*
NHR (%)	38.40 ± 0.47	34.71 ± 1.25	0.003*
TG, mmol/L	1.16 ± 0.03	1.08 ± 0.08	0.523
LDL-C, mmol/L	2.45 ± 0.03	2.58 ± 0.07	0.069
FBG, mmol/L	5.15 ± 0.02	5.15 ± 0.05	0.713

SBP, systolic blood pressure; DBP, diastolic blood pressure; BMI, body mass index; WBC, white blood cells; LHR, lymphocyte percentage to HDL-C ratio;MHR, monocyte percentage to HDL-C ratio; NHR, neutrophil percentage to HDL-C ratio; TG, triglyceride; LDL-C, low-density lipoprotein cholesterol; FBG, fasting blood glucose; Data are presented in mean ± SD for continuous measures and n (%) for categorical measures. Significance in Chi-Squared test for proportions or t-test for continuous measurements; *: *P* < 0.05.

### Vitamin D deficiency–associated factors

To eliminate the influence of confounding factors, statistically significant variables in the univariate logistic regression analysis were input as independent variables and vitamin D deficiency was used as the dependent variable in the logistic regression analysis. Consequently, gender (*β*=0.334, *P* = 0.003), age (*β* = 0.919, *P* < 0.001), LHR (*β* = 1.052, *P* = 0.008), MHR (*β* = 1.646, *P* < 0.001), NHR (*β* = 1.040, *P* = 0.004), LDL-C (*β* = 0.709, *P* = 0.092) were further incorporated into the multivariate logistic regression analysis. In the subsequent analysis, males exhibited a higher risk of vitamin D deficiency compared to females (*β* = 0.383, *P* = 0.026). Vitamin D deficiency risk decreased with age (*β* = 0.910, *P* < 0.001). Additionally, elevated values of MHR were associated with an increased risk of vitamin D deficiency (β = 1.530, *P* = 0.019). (Table 2)

**Table 2:** Vitamin D deficiency–associated factors

Variables	Univariate logistic regression analysis		Multivariate logistic regression analysis	
	*β* (95% *CI*)	*P*	β (95% CI)	*P* ^b^
Gender^a^	0.334 (0.162, 0.690)	0.003^*^	0.383 (0.164, 0.891)	0.026^**^
Age, year	0.919 (0.898, 0.939)	< 0.001^*^	0.910 (0.886, 0.935)	< 0.001^**^
WBC, cells/10^9^ L	1.144 (0.958, 1.368)	0.138	-	-
LHR (%)	1.052 (1.013, 1.093)	0.008 ^*^	0.979 (0.932, 1.029)	0.412
MHR (%)	1.646 (1.276, 2.125)	< 0.001^*^	1.530 (1.071, 2.185)	0.019^**^
NHR (%)	1.040 (1.012, 1.068)	0.004 ^*^	1.005 (0.973, 1.039)	0.747
TG, mmol/L	1.175 (0.808, 1.708)	0.399	-	-
LDL-C, mmol/L	0.709 (0.476, 1.058)	0.092 ^*^	1.173 (0.689, 2.000)	0.556
FBG, mmol/L	1.019 (0.586, 1.772)	0.948	-	-
BMI, kg/m^2^	1.033 (0.947, 1.126)	0.462	-	-
Smoking	0.886 (0.331, 2.373)	0.810	-	-

BMI, body mass index; WBC, white blood cells; LHR, lymphocyte percentage to HDL-C ratio;MHR, monocyte percentage to HDL-C ratio; NHR, neutrophil percentage to HDL-C ratio; TG, triglyceride; LDL-C, low-density lipoprotein cholesterol; FBG, fasting blood glucose; ^a^: The male was served as the reference group. ^*b*^: Variables with a *P*-value < 0.1 in the univariate analysis were included in the multivariate logistic regression analysis; ^*^: *P* < 0.1, **: *P* < 0.05.

### Vitamin D level–associated factors

Vitamin D was considered a continuous variable to identify factors influencing vitamin D levels. Initially, a univariate linear regression analysis was conducted, and independent variables with a *P*-value < 0.1 (gender, age, LHR, MHR, NHR, TG, and LDL-C) were included in the multivariate linear regression analysis. The results revealed that there were significant associations observed between age and vitamin D levels, with an increase in vitamin D levels as age advanced (*β* = 0.342, *P* < 0.001). Conversely, a decrease in triglyceride levels was observed with increasing vitamin D levels (*β*=-1.327, *P* = 0.010). A reverse correlation between MHR and vitamin D levels demonstrated a marginal trend toward significance (β=-0.581, *P* = 0.052). (Table 3)

**Table 3:** Vitamin D level–associated factors

Variables	Univariate linear regression analysis		Multivariate linear regression analysis	
	*β* (95% *CI*)	*P*	*β* (95% *CI*)	*P* ^*b*^
Gender ^a^	2.079 (0.685, 3.474)	0.004^*^	1.203 (-0.187,2.593)	0.090
Age, year	0.332 (0.278, 0.385)	0.035 ^*^	0.342 (0.286, 0.398)	< 0.001^**^
WBC, cells/10^9^ L	-0.260 (-0.687, 0,167)	0.233	-	-
LHR (%)	-0.155 (-0.240, -0.069)	< 0.001^*^	0.024 (-0.070, 0.117)	0.616
MHR (%)	-0.953 (-1.440, -0.466)	< 0.001^*^	-0.581(-1.169,0.006)	0.052
NHR (%)	-0.092 (-0.152, -0.031)	0.003 ^*^	0.013(-0.054, 0.079)	0.709
TG, mmol/l	-0.869 (-1.722, -0.017)	0.046 ^*^	-1.327(-2.329, -0.325)	0.010 ^**^
LDL-C, mmol/l	1.052 (0.007, 2.098)	0.048^*^	0.397 (-0.716, 1.510)	0.484
FBG, mmol/L	0.792 (-0.609, 2.194)	0.267	-	-
BMI, kg/m^2^	-0.012 (-0.233, 0.208)	0.913	-	-
Smoking	-0.230 (-2.828, 2.369)	0.862	-	-

BMI, body mass index; WBC, white blood cells; LHR, lymphocyte percentage to HDL-C ratio;MHR, monocyte percentage to HDL-C ratio; NHR, neutrophil percentage to HDL-C ratio; TG, triglyceride; LDL-C, low-density lipoprotein cholesterol; FBG, fasting blood glucose; ^a^: The male was served as the reference group. ^*b*^: Variables with a *P*-value < 0.1 in the univariate analysis were included in the multivariate linear regression analysis; ^*^: *P* < 0.1, **: *P* < 0.05.

### Correlation analysis between vitamin D levels and age, BMI across different genders

**Fig. 1:**
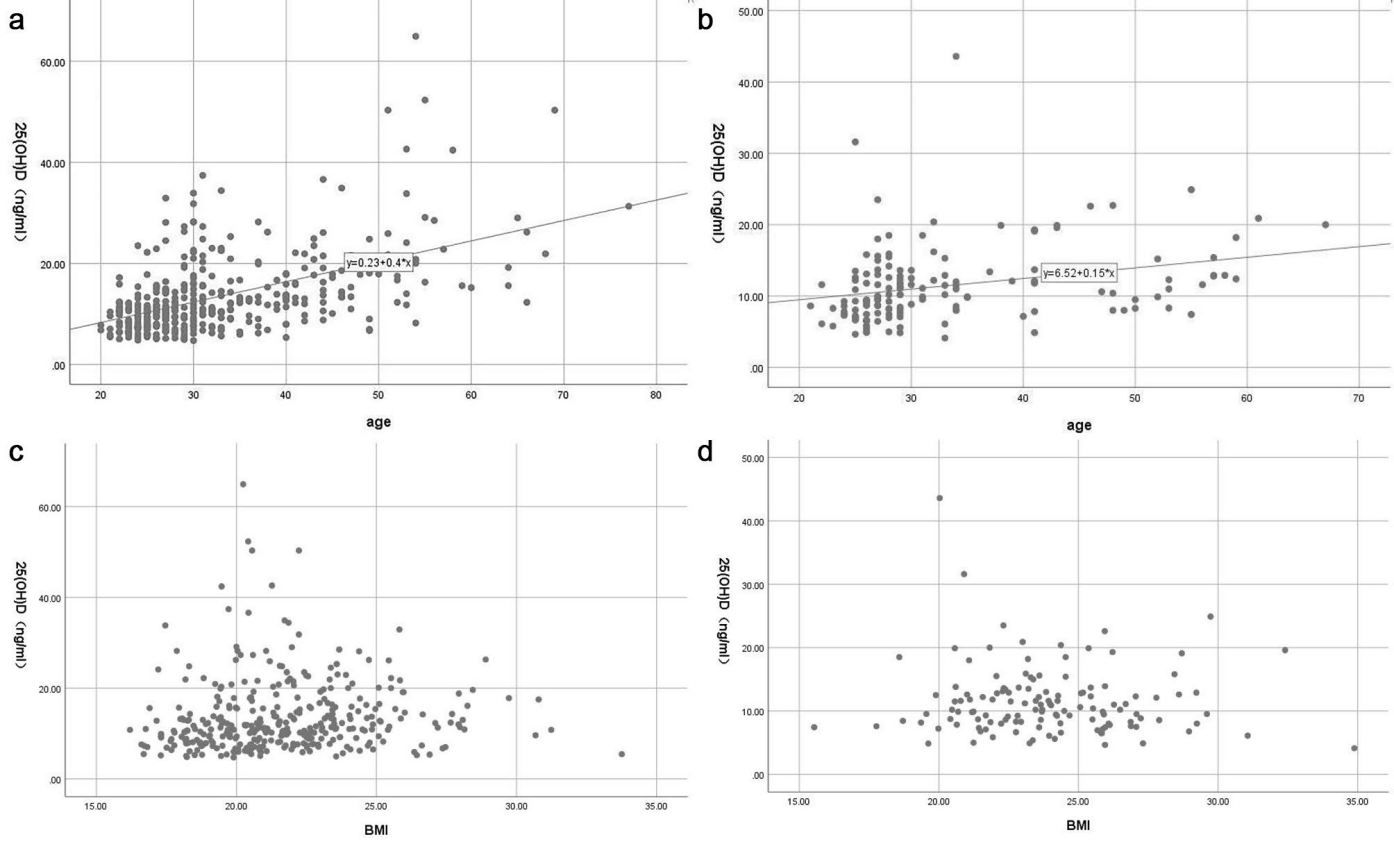
Correlation analysis between vitamin D levels and age, BMI across different genders. **a:** Correlation between vitamin D levels and age for female; **b:** Correlation between vitamin D levels and age for male; **c:** Correlation between vitamin D levels and BMI for female; **d:** Correlation between vitamin D levels and BMI for male.

Based on Pearson correlation analysis, there was a positive correlation between vitamin D levels and age in both female (*r* = 0.524, *P* < 0.001) and male (*r* = 0.290, *P* < 0.001) groups, suggesting that as age increased, vitamin D levels also increased. Pearson correlation analysis showed that there was no significant association between vitamin D levels and BMI ((*P*_male_=0.479, *P*_female_=0.217)). (Fig. 1).

## Discussion

Vitamin D was first defined as a vitamin in the 20th century, and nowadays it is recognized as a prohormone with a vital role in the responses of the immune [17]. As previously mentioned, vitamin D deficiency has been linked to several diseases. In recent years, the study of vitamin D has become a research hotspot due to its clinical significance. The prevalence of vitamin D deficiency is high in the general population [18]. However, universal screening for vitamin D is not cost-effective compared to routine blood and lipid tests. Therefore, it is crucial to identify a reliable predictor to identify individuals at high risk for vitamin D deficiency.

In this study, we investigated the association between serum 25 (OH)D and MHR among Chinese medical staff. Higher values of MHR were found to be associated with an increased risk of vitamin D deficiency. MHR emerged as an independent factor influencing the occurrence of vitamin D deficiency. The results demonstrated that MHR was inversely associate with vitamin D levels.

MHR is a new indicator of inflammation and oxidative stress [11]. MHR was independently and significantly associated with ST-elevation myocardial infarction [19]. A newly published retrospective study from China showed that MHR was an independent risk factor for in-stent restenosis in premature coronary heart disease patients and had certain prognostic value [20]. Some research revealed the relationship with other diseases (diabetes mellitus, polycystic ovary syndrome, obstructive sleep apnea syndrome, etc.) [2, 21, 22].

Vitamin D has been linked to inflammation in several studies. It played an essential role in macrophage differentiation and modulated the host response against inflammation [23]. Vitamin D levels can influence the development and severity of acute respiratory infections, such as influenza and acute pneumonia [24, 25]. Results from a meta-analysis involving 46 randomized controlled trials showed that vitamin D supplementation demonstrated a protective effect against acute respiratory tract infections [26]. Furthermore, vitamin D deficiency was associated with the severity/mortality of COVID-19, and it’s necessary to supplement vitamin D in COVID-infected individuals [27].

Inflammation may be an important mediator in the association between vitamin D and MHR. An inverse association between vitamin D and serum inflammatory markers, including C-reactive protein, interleukin-6, and tumor necrosis factor α, had been recently discovered [5, 6]. However, to date, little research has been done on the association between 25 (OH)D and MHR [28, 29]. The inverse association we observed is consistent with a study conducted in Qatar [28]. However, that study just included adults between the ages of 18 and 40. In contrast to previous investigations, our study demonstrated a decreased incidence of vitamin D deficiency in the elderly population [30, 31]. The results of other scholars also align with ours [32]. One possible explanation is that young individuals or medical professionals may spend more time indoors, such as in their work environment. A systematic review indicated that individuals with indoor occupations, including shift workers, health care workers, and doctors, were at a higher risk for vitamin D deficiency, likely due to lifestyle factors such as reduced sun exposure [33]. Men are at a higher risk of vitamin D deficiency, as they are more likely to be current smokers and less likely to take vitamin D supplements [34]. These factors suggest that there may be gender-specific reasons for the prevalence of vitamin D deficiency in men.

Monocytes contribute directly to immune defense against microbial pathogens [35]. HDL-C possesses well-established anti-inflammatory and antioxidant properties [36]. The inverse association between vitamin D and MHR may be elucidated by several underlying mechanisms. Firstly, vitamin D can reduce monocyte activation by down-regulating adhesion molecules such as PSGL-1, β(1) -integrin, and β(2) -integrin [37]. Additionally, vitamin D suppressed the expression of receptor proteins and mRNA in human monocytes in a time- and dose-dependent fashion [38].

It should be noted that this study has some limitations. Firstly, because this was a cross-sectional study, making causal connections between vitamin D deficiency risk and MHR should be done with caution. Secondly, although multiple variables were adjusted, other potential confounding factors such as socioeconomic variables, lifestyle habits (including dietary habits and physical activity, etc.), and medication use (including a history of vitamin D supplementation, etc.) may still be present. Thirdly, the study population comprised medical staff with a specific occupational context, and the sample size was relatively small, thus limiting the generalizability of the results. Therefore, future multicenter studies with larger sample sizes are required to extend these findings.

## Conclusions

Vitamin D is inversely associated with the monocyte to HDL-C ratio among young medical staff in Chengdu, China.

## Data Availability

The data that support the findings of this study are available from the Affiliated Hospital of Chengdu Medical University, but restrictions apply to the availability of these data, which were used under license for the current study and are therefore not publicly available. Data are, however, available from the authors (Jia Pan, panjiapanda@163.com) upon reasonable request and with permission of the Affiliated Hospital of the Chengdu Medical University.
